# Supplementary material for: Functional validation of human SK channels variants causing NEDMAB and Zimmermann–Laband syndrome-3 in C. elegans
Source: Brain Commun. 2025 Sep 16;7(5):fcaf351. doi: 10.1093/braincomms/fcaf351 (PMC12484442; doi:10.1093/braincomms/fcaf351)
Supplement: fcaf351_Supplementary_Data [file fcaf351_supplementary_data.zip › Supplementary_material.pdf]

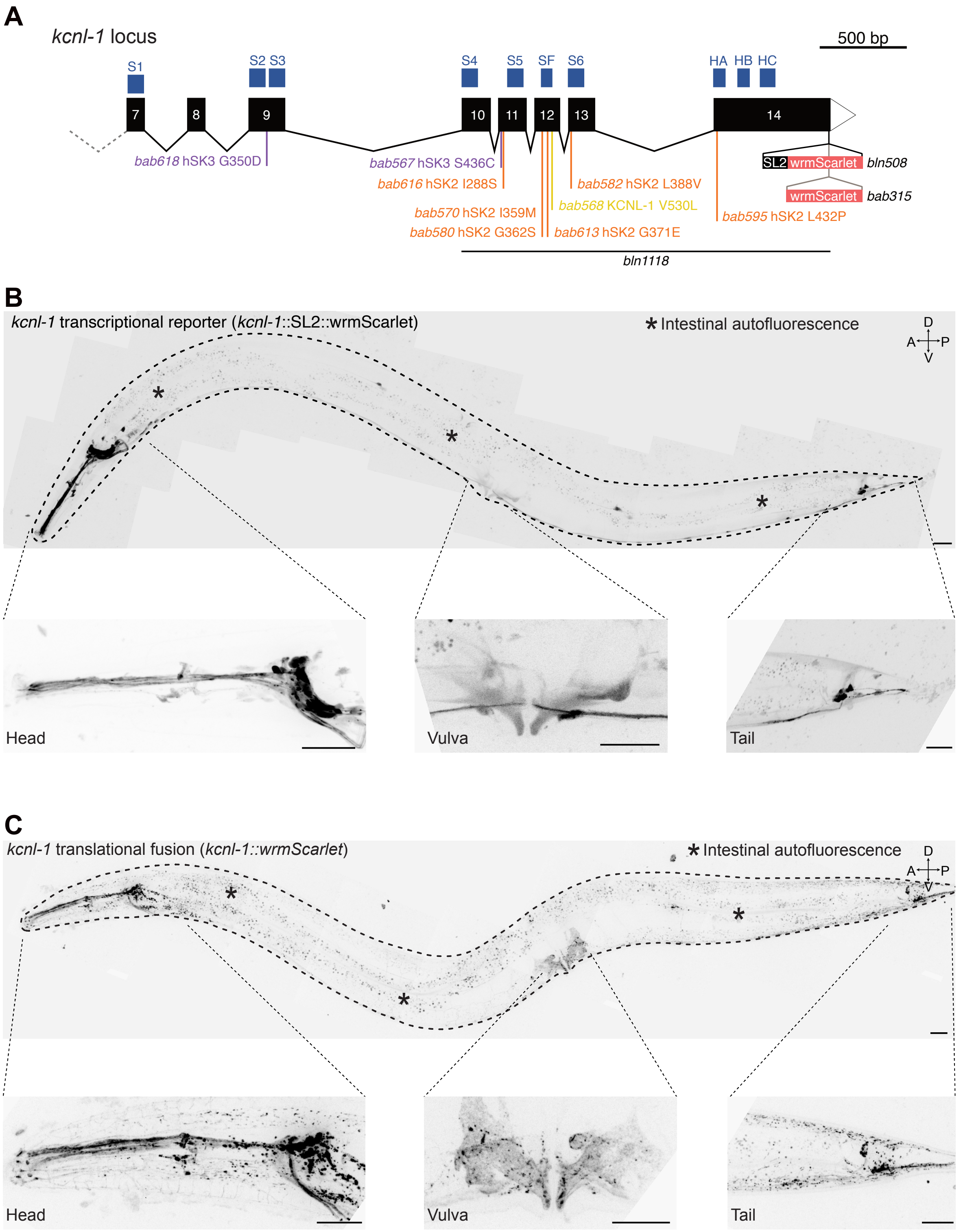

Supplementary Figure 1. *kcnl-1* gene structure and translational reporter.

A. *kcnl-1* gene structure. Common exons (7 to 14) for six isoforms are shown in black. Blue boxes indicate the approximate positions of coding regions for transmembrane segments (S1-S6), the helices A, B and C (HA, HB, HC), and the selectivity filter (SF). The wrmScarlet coding sequence was inserted in frame before the stop codon of the last exon. ZLS3-hSK3 patient variants are indicated in

purple, NEDMAB-hSK2 in orange, and the natural gain-of-function mutation in yellow. Alleles are indicated in italics. The extent of the *bln1118* deletion allele is indicated by a black bar.

B. Lateral view of a young adult stage animal for the fluorescent transcriptional reporter line *kcnl-1(bln508)*. Magnified views of the head, vulval region, and tail. Worm outline, dashed line. Asterisks indicate intestinal autofluorescence. Scale bars, 20 µm.

C. Lateral view of a young adult stage animal for the fluorescent translational knock-in line *kcnl-1(bab315)*. Magnified views of the head, vulval region, and tail. Worm outline, dashed line. Asterisks indicate intestinal autofluorescence. Scale bars, 20 µm.

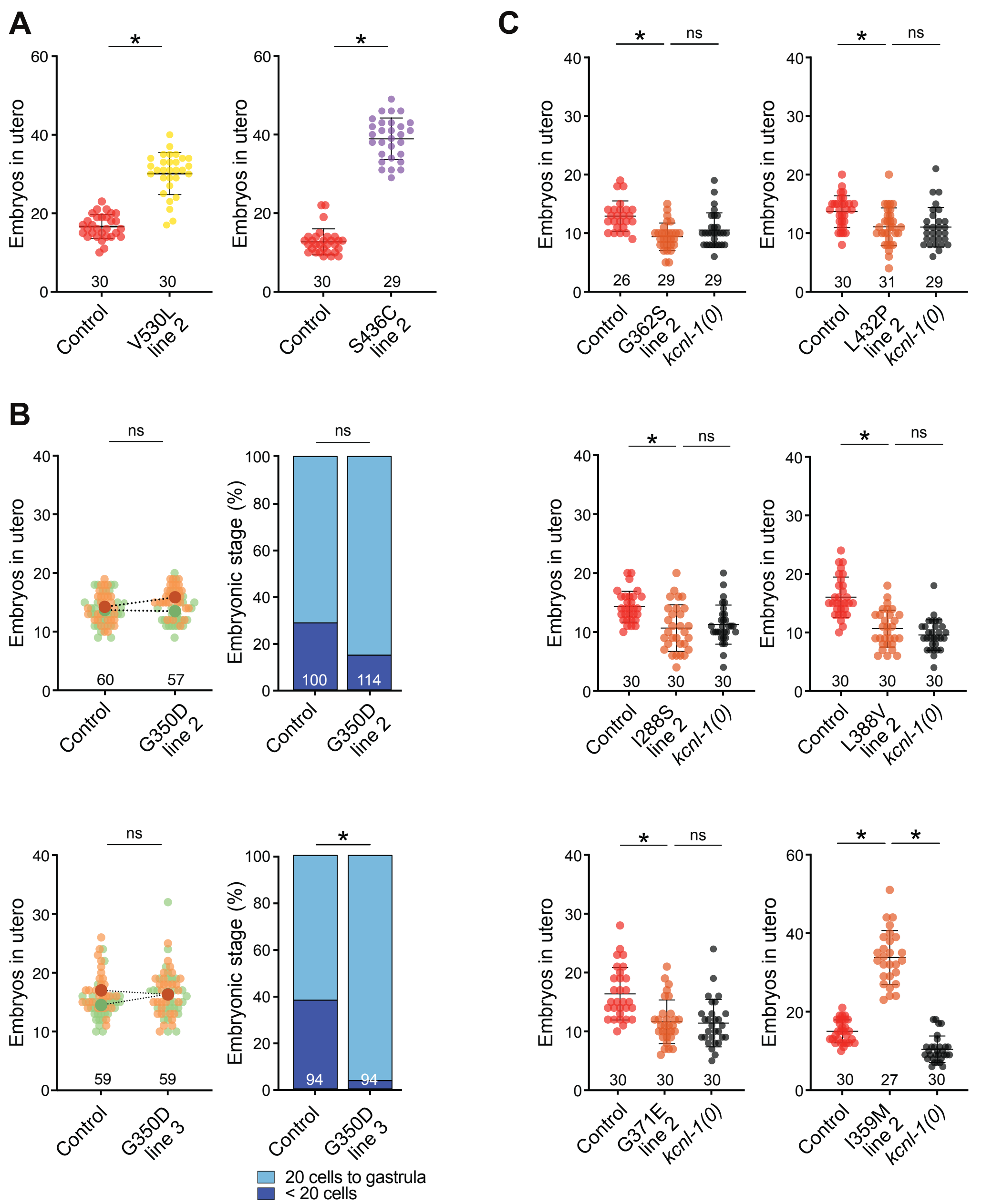

Supplementary Figure 2. Egg-laying phenotypes of additional independent *kcnl-1* alleles.

A. *In utero* egg accumulation for additional independent mutant lines. Dots represent individual animals. Control N=30 V530L line 2 N=30; Control N=30, S436C line 2 N=29. Mann Whitney, * $p < 0.05$.

B. *Left panel*, *in utero* egg accumulation for two independent G350D lines. Small salmon and green dots represent individual animals coming from two replicates. Large dots indicate the mean. Control N=60 G350D line 2 N=57; Control N=59, G350D line 3 N=59. Mann-Whitney test, ns $p > 0.05$.

Right panel, distribution of developmental stages of embryos laid over 30 min by 30 hermaphrodite animals. Control N=100, G350D line 2 N=114; Control N=94, G350D line 3 N=94. Fisher's exact test with Bonferroni correction, ns $p > 0.05$, * $p < 0.05$.

C. *In utero egg accumulation* from independent alleles for NEDMAB-hSK2 variants. Dots represent individual animals. Control N=26, G362S line 2 N=29, *kcnl-1(0)* N=29; Control N=30, L432P line 2 N=31, *kcnl-1(0)* N=29; Control N=30, I288S line 2 N=30, *kcnl-1(0)* N=30; Control N=30, L388V line 2 N=30, *kcnl-1(0)* N=30; Control N=30, G371E line 2 N=30, *kcnl-1(0)* N=30; Control N=30, I359M line 2 N=27, *kcnl-1(0)* N=30. Kruskal-Wallis test for I288S, G362S, L388V and L432P, ns $p > 0.05$, * $p < 0.05$. Ordinary One-Way ANOVA for G371E and I359M, ns $p > 0.05$, * $p < 0.05$.

Raw data are available in Appendix 1 of the Supplementary material.

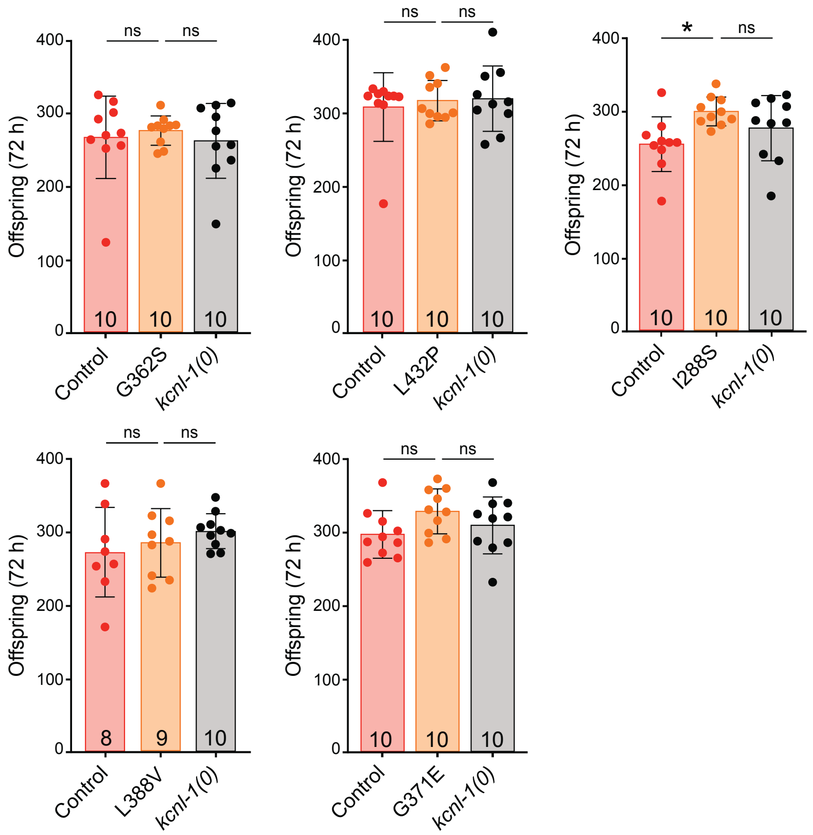

Supplementary Figure 3. I288S, G371E, G362S, L432P, L388V and null mutations do not affect brood size.

Brood size over 72 hours for control animals or mutants carrying the patient variants G362S, L432P, L388V, I228S or G371E. Each dot represents the offspring of one animal. Control N=10, G362S N=10, *kcnl-1(0)* N=10; Control N=10, L432P N=10, *kcnl-1(0)* N=10; Control N=10, I288S N=10, *kcnl-1(0)* N=10; Control N=8, L388V N=9, *kcnl-1(0)* N=10; Control N=10, G371E N=10, *kcnl-1(0)* N=10. Kruskal-Wallis tests were performed for G362S and L432P, while Ordinary One-Way ANOVA were performed for G371E, I288S and L388V. ns $p > 0.05$, * $p < 0.05$.
Raw data are available in Appendix 1 of the Supplementary material.

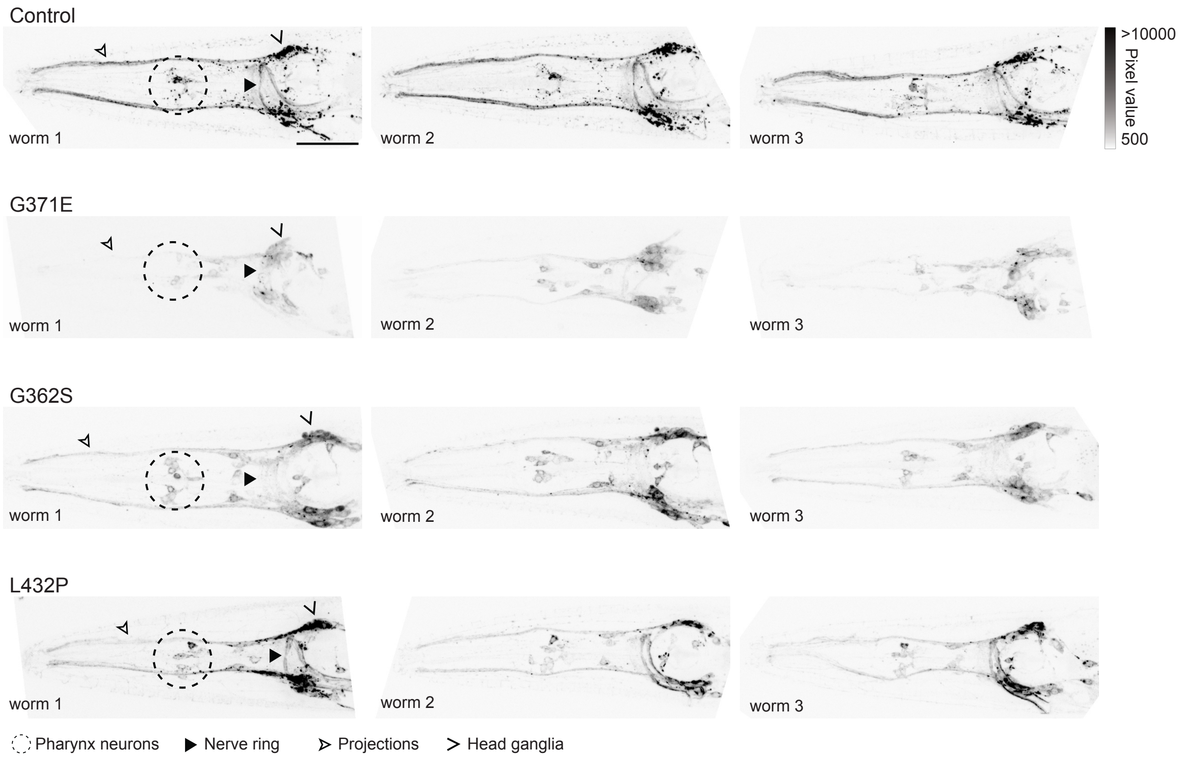

Supplementary Figure 4. Supplementary images for KCNL-1 fluorescence profiles in the head of control animals and of G371E, G362S, and L432P NEDMAB-hSK2 variants.

Representative images of three different animals showing KCNL-1 localization in the head of control animals and G371E, G362S and L432P mutants. wrmScarlet fluorescent translational reporter is visible in several neurons of the pharynx (dashed circle), the head ganglia (open arrowhead), projections to the tip of the nose (empty arrowhead), and the nerve ring (arrowhead). Dorso-ventral views. Scale bar, 20 μm.

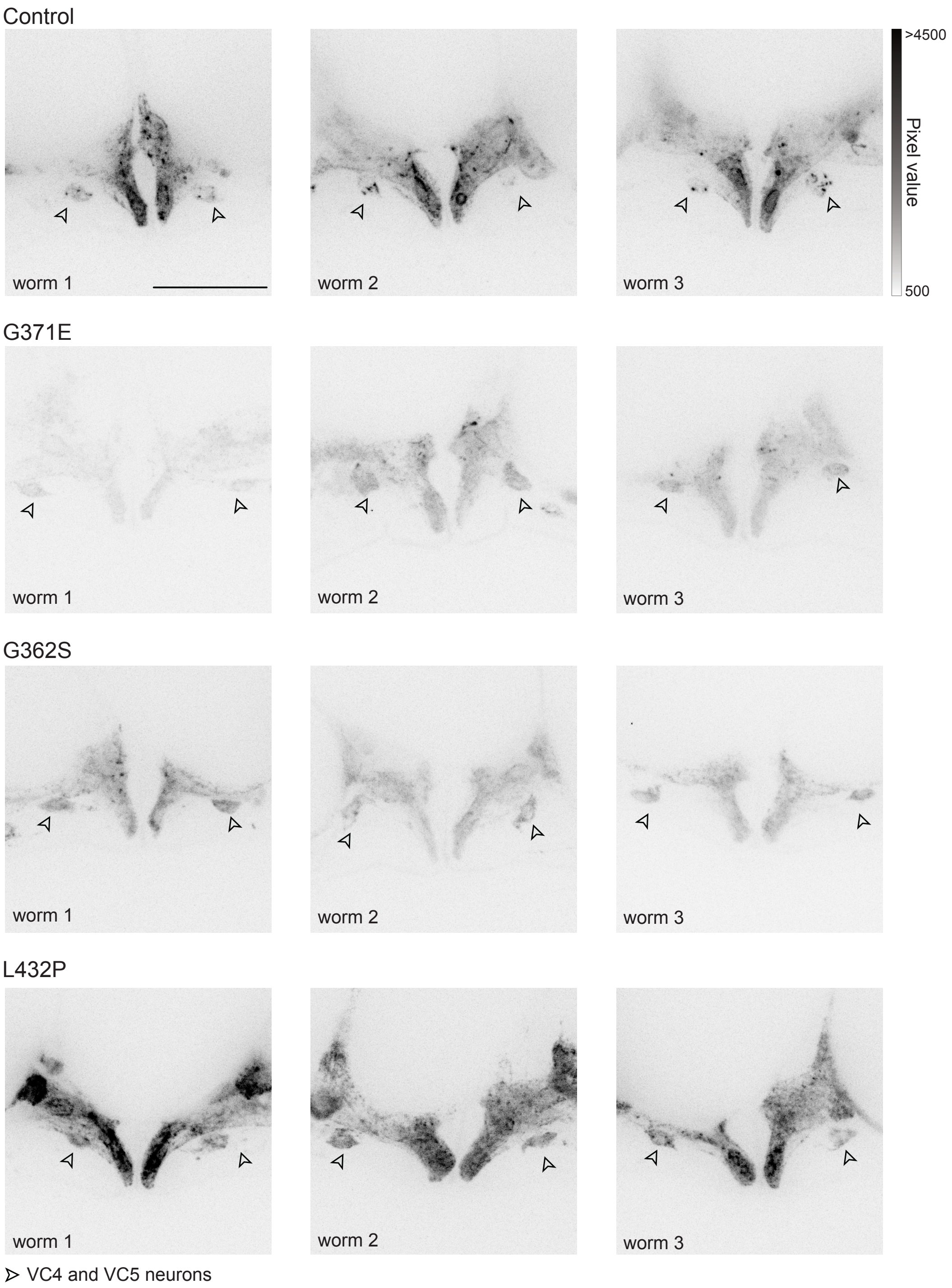

Supplementary Figure 5. Supplementary images for KCNL-1 fluorescence profiles in the egg-laying apparatus of control animals and of G371E, G362S, and L432P NEDMAB-hSK2 variants.

Representative images of three different animals showing KCNL-1 localization in the egg-laying apparatus of control animals or mutants carrying the G371E, G362S, or L432P NEDMAB-hSK2 variants based on wrmScarlet fluorescent translational reporter. Cell bodies of VC4 and VC5 motor neurons are indicated by empty arrowheads. Lateral views; anterior, left; ventral, down. Scale bar, 20 μm.

Supplementary Table 1 List of strains and associated alleles

Supplementary Table 2 List of oligonucleotides (crRNA, Repair template and PCR primers) for strain generation and validation

Supplementary Table 3 Correspondence between patient variants in hSK2/hSK3 and KCNL-1

Supplementary Table 4 Statistics

Appendix 1 Raw data

Appendix 2 Python code for statistical analysis of embryonic stage distributions

Appendix 1

Raw data presented in figure 1C

Embryos in utero - Control vs *kcnl-1(0)*

First repetition		Second repetition	
Control (JIP3105)	*kcnl-1(0)* JIP3131	Control (JIP3105)	G350D (JIP3127)
13	9	11	9
13	14	10	8
16	12	16	14
13	11	17	10
10	8	12	12
11	10	12	8
12	9	18	7
13	6	13	11
10	9	9	7
18	12	15	6
10	13	13	13
12	8	11	9
16	12	12	8
14	8	9	9
16	9	18	9
13	11	11	12
15	10	16	10
16	9	8	8
15	8	14	12
9	7	16	10
13	10	17	9
14	11	14	10
16	8	14	7
12	9	12	9
12	13	18	11
16	6	16	9
13	10	13	5
	13	11	12
	7	10	10
	8		

Mann Whitney test
P value <0.0001

Embryonic stage (%) - Control vs *kcnl-1(0)*

	<20 cells	20 cells to gastrula
Control (JIP3105)	29	71
kcnl-1(0) (JIP3131)	48	53

Fisher's Exact + Bonferroni correction

Category	P value	Correction
<20 cells	0.0089	0.0178
20 cells to gastrula	0.0089	0.0178

Raw data presented in figure 2C and 2D

Embryos in utero - V530L and ZLS variants

First repetition		Second repetition	
Control (JIP3105)	V530L (JIP3106)	Control (JIP3105)	V530L (JIP3106)
12	32	24	50
15	37	23	45
19	31	21	43
14	23	30	23
17	30	19	29
19	35	15	34
20	26	28	44
19	28	21	46
20	25	18	36
15	20	22	36
15	35	26	57
22	32	21	31
16	44	19	38
22	22	16	30
19	30	21	36
16	18	21	29
16	18	19	31
20	26	22	41
10	34	23	37
13	20	16	41
17	34	21	52
17	20	21	39
22	41	22	37
13	38	23	26
22	27	22	31
14	30	24	36
13	26	14	52
19	40	21	40
11	26	20	49
	22	25	37

Mann Whitney test
P value <0.0001

First repetition		Second repetition	
Control (JIP3105)	S436C (JIP3108)	Control (JIP3105)	S436C (JIP3108)
19	37	12	47
18	40	21	43
15	40	16	57
13	36	21	45
16	38	12	46
28	40	23	42
19	38	20	46
14	36	21	42
18	35	28	33
14	32	24	32
14	31	20	45
14	50	20	59
19	42	17	48
18	39	12	60
17	39	22	56
25	42	21	36
13	41	32	38
16	36	18	58
15	39	35	38
16	39	26	47
12	39	21	56
16	39	25	42
20	25	20	43
15	37	24	59
18	18	18	47
21	39	27	51
19	42	20	49
22	19	13	44
15	40	18	28
16	31	15	44

Mann Whitney test
P value <0.0001

Raw data presented in figure 2E

Embryos in utero - V530L and ZLS variants

First repetition		Second repetition	
Control (JIP3105)	G350D (JIP3127)	Control (JIP3105)	G350D (JIP3127)
18	18	21	30
20	20	16	20
19	22	20	26
16	21	18	23
18	21	30	32
13	17	20	18
25	34	27	29
19	16	38	24
15	19	16	30
17	20	16	30
17	19	19	17
14	26	25	31
15	20	12	24
22	19	27	26
20	16	20	21
15	20	21	29
16	20	24	22
18	21	32	29
16	19	13	22
16	35	19	14
18	20	14	21
20	16	28	25
16	20	20	17
19	19	16	10
22	14	18	26
27	23	20	14
21	22	21	19
19	21	21	22
21	23	15	27
15	19	17	33

Mann Whitney test
P value 0.0012

Raw data presented in figure 2C, 2D, 2E

Embryonic stage (%) - V530L and ZLS variants

	<20 cells	20 cells to gastrula	post gastrula
**Control (JIP3105)**	34	68	0
**V530L (JIP3106)**	0	78	25

Fisher's Exact + Bonferroni correction

Category	P value	Correction
<20 cells	0.0000	0.0000
20 cells to gastrula	0.1673	0.5018
post gastrula	0.0000	0.0000

	<20 cells	20 cells to gas	post gastrula
**Control (JIP3105)**	38	62	0
**S436C (JIP3108)**	0	63	40

Fisher's Exact + Bonferroni correction

Category	P value	Correction
<20 cells	0.0000	0.0000
20 cells to gastrula	1.0000	1.0000
post gastrula	0.0000	0.0000

	<20 cells	20 cells to gas	post gastrula
**Control (JIP3105)**	50	68	0
**G350D (JIP3127)**	10	97	0

Fisher's Exact + Bonferroni

Category	P value	Correction
<20 cells	0.0000	0.0000
20 cells to gastrula	0.0000	0.0000

Raw data presented in figure 2C, 2D, 2E

Brood size - V530L and ZLS variants

Control (JIP3105)	V530L (JIP3106)
276	172
	216
236	222
260	187
267	102
278	38
283	182
310	204
259	173
250	171

Mann Whitney test
P value <0.0001

Control (JIP3105)	S436C (JIP3108)
350	254
	231
303	48
343	199
195	244
145	236
338	217
326	72
269	211
352	202

Mann Whitney test
P value = 0.0172

Control (JIP3105)	G350D (JIP3127)
316	351
290	293
285	252
340	268
331	288
345	241
324	331
323	288
340	322
	275

Mann Whitney test
P value = 0.0559

Raw data presented in figure 3C

Embryos in utero - NEDMAB variants

First repetition			Second repetition		
**Control (JIP3105)**	**G362S (JIP3121)**	***kcnl-1(0)* (JIP3131)**	**Control (JIP3105)**	**G362S (JIP3121)**	***kcnl-1(0)* (JIP3131)**
21	17	11	27	21	18
17	14	15	30	11	15
23	13	17	13	14	27
16	12	13	24	5	16
23	16	17	18	18	17
17	10	15	27	20	19
21	20	12	26	17	17
21	17	15	24	16	9
21	5	15	18	13	12
23	7	10	25	18	13
27	12	10	24	11	11
21	14	11	27	13	17
15	11	14	19	13	9
15	11	11	12	8	17
22	11	17	14	20	14
23	16	13	15	14	11
13	8	13	28	13	28
28	8	12	25	19	15
21	7	9	12	13	13
22	8	17	7	15	14
19	16	12	23	14	11
16	11	9	29	10	19
20	13	11	20	17	16
20	6	6	18	19	15
17	17	7	25	16	16
16	17	11	22	10	29
19	26	10	18	10	12
16	8	15	16	17	14
27	9	9	19	17	18
24	11	18	21	20	11

Kruskal-Wallis test P value <0.0001

Raw data presented in figure 3D

Embryos in utero - NEDMAB variants

First repetition			Second repetition		
**Control (JIP3105)**	**L432P (JIP3124)**	***kcnl-1(0)*** **(JIP3131)**	**Control (JIP3105)**	**L432P (JIP3124)**	***kcnl-1(0)*** **(JIP3131)**
17	8	12	16	19	14
11	18	8	30	13	15
22	17	12	17	19	16
16	11	17	16	11	12
19	11	10	11	16	17
27	13	10	21	16	15
16	16	13	17	20	11
15	17	9	25	13	14
17	19	12	18	10	17
15	14	12	16	12	19
26	16	12	24	8	9
18	14	10	17	12	12
16	13	23	22	14	11
17	10	10	23	12	18
23	22	25	17	14	15
14	12	14	22	11	18
9	13	16	11	16	14
17	14	27	18	12	13
24	13	14	23	12	13
19	8	10	17	11	10
19	12		19	13	13
16	16		21	13	13
21	12		21	15	15
12	13		20	15	6
14	17		20	15	19
15	21		17	13	15
16	17		15	15	8
17	13		20	9	27
17	15		22	12	10
12	22		16	15	11

Kruskal-Wallis test P value <0.0001

Raw data presented in figure 3E

Embryos in utero - NEDMAB variants

First repetition			Second repetition		
Control (JIP3105)	I288S (JIP3114)	*kcnl-1(0)* (JIP3131)	Control (JIP3105)	I288S (JIP3114)	*kcnl-1(0)* (JIP3131)
16	9	13	22	23	14
13	9	12	28	19	13
16	8	15	30	20	18
14	14	13	18	14	14
18	7	10	26	20	26
21	10	7	28	19	15
23	13	15	25	15	17
14	5	13	16	34	24
20	14	10	23	22	13
24	13	17	22	22	17
20	9	11	29	16	16
14	11	8	27	11	18
17	9	8	21	25	21
16	10	9	27	21	16
12	10	7	20	13	16
13	9	15	24	17	25
16	8	5	22	25	23
23	5	8	27	22	22
18	9	15	22	15	23
15	7	9	27	23	19
18	12	5	24	20	29
16	9	18	26	12	14
10	12	9	31	21	8
22	10	10	30	12	17
21	13	12	21	17	12
23	9	10	27	15	22
17	11	9	29	11	27
16	13	13	23	15	24
17	10	8	24	17	20
19	11	6	27	19	17

Kruskal-Wallis test P value <0.0001

Raw data presented in figure 3F

Embryos in utero - NEDMAB variants

First repetition			Second repetition		
**Control (JIP3105)**	**L388V (JIP3123)**	***kcnl-1(0)* (JIP3131)**	**Control (JIP3105)**	**L388V (JIP3123)**	***kcnl-1(0)* (JIP3131)**
20	22	16	12	13	11
12	6	22	14	10	8
20	18	18	16	12	6
16	20	18	18	17	9
19	15	15	18	12	11
15	19	23	12	10	8
24	17	14	11	10	9
16	16	8	11	12	10
25	18	8	14	10	7
18	20	17	12	11	13
11	18	9	16	9	17
13	18	11	14	16	8
22	15	13	12	9	11
17	16	11	13	7	10
13	18	1	16	8	14
23	16	21	10	11	5
18	15	17	14	12	7
10	14	11	12	11	15
22	18	23	12	11	8
21	28	13	12	8	7
18	6	18	13	14	9
16	14	14	12	9	6
14	18	14	10	11	8
22	13	11	10	14	4
28	16	14	10	12	9
12	16	11	13	10	11
19	15	5	15	10	11
16	18	15	13	12	5
18	15	12	16	10	15
14	13	22		10	7
				8	

Kruskal-Wallis test P value Control vs. L388V 0,0906;
P value L388V vs. *kcnl-1(0)* 0,0672

Raw data presented in figure 3G

Embryos in utero - NEDMAB variants

First repetition			Second repetition		
Control (JIP3105)	G371E (JIP3110)	*kcnl-1(0)* (JIP3131)	Control (JIP3105)	G371E (JIP3110)	*kcnl-1(0)* (JIP3131)
19	21	18	17	13	10
14	18	17	16	11	12
21	16	20	17	12	10
13	19	16	11	12	9
19	12	17	16	7	12
13	16	17	10	9	5
13	13	16	19	13	12
24	19	14	15	13	7
19	18	17	15	7	9
18	16	20	11	10	5
18	14	9	18	10	13
17	15	16	23	14	10
16	16	19	13	7	6
23	7	12	11	15	11
22	13	18	12	11	8
21	11	10	22	10	6
12	11	11	13	13	7
24	17	8	13	17	5
11	10	12	16	9	6
16	9	14	15	8	14
21	9	7	15	11	10
19	8	18	16	10	11
22	11	14	10	8	6
18	12	22	14	11	12
17	15	20	12	7	10
16	17	12	13	7	77
19	13	16	19	13	12
18	16	19	12	8	9
21	14	14	12	9	
23	14	18	15	8	

Kruskal-Wallis test P value <0.0001

Raw data presented in figure 3C, 3D and 3E

Embryonic stage (%) - NEDMAB variants

	<20 cells	20 cells to gastrula
**Control (JIP3105)**	41	67
**G362S (JIP3121)**	58	48
**kcnl-1(0) (JIP3131)**	57	52

			**Control vs G362S**	**Control vs *kcnl-1 (0)***	***kcnl-1 (0)* vs G362S**
**Fisher Exact + Bonferroni correction**	**< 20 cells**	P value	0.0196	0.0409	0.7849
		Correction	0.0391	0.0817	1.0000
	**20 cells to gastrula**	P value	0.0196	0.0409	0.7849
		Correction	0.0391	0.0817	1.0000

	<20 cells	20 cells to gastrula
**Control (JIP3105)**	28	74
**L432P (JIP3124)**	51	55
**kcnl-1(0) (JIP3131)**	54	46

			**Control vs L432P**	**Control vs *kcnl-1 (0)***	***kcnl-1 (0)* vs L432P**
**Fisher Exact + Bonferroni correction**	**< 20 cells**	P value	0.0027	0.0002	0.4069
		Correction	0.0053	0.0004	0.8139
	**20 cells to gastrula**	P value	0.0027	0.0002	0.4069
		Correction	0.0053	0.0004	0.8139

	<20 cells	20 cells to gastrula
**Control (JIP3105)**	33	73
**I288S (JIP3114)**	64	48
**kcnl-1(0) (JIP3131)**	53	48

			**Control vs I288S**	**Control vs *kcnl-1 (0)***	***kcnl-1 (0)* vs I288S**
**Fisher Exact + Bonferroni correction**	**< 20 cells**	P value	0.0001	0.0020	0.5814
		Correction	0.0003	0.0040	1.0000
	**20 cells to gastrula**	P value	0.0001	0.0020	0.5814
		Correction	0.0001	0.0040	1.0000

Raw data presented in figure 3F and 3G

Embryonic stage (%) - NEDMAB variants

	<20 cells	20 cells to gastrula
**Control (JIP3105)**	35	66
**L388V (JIP3123)**	63	47
**kcnl-1(0) (JIP3131)**	58	44

			**Control vs L388V**	**Control vs *kcnl-1 (0)***	***kcnl-1 (0)* vs L388V**
**Fisher Exact + Bonferroni correction**	**< 20 cells**	P value	0.0014	0.0019	1.0000
		Correction	0.0029	0.0038	1.0000
	**20 cells to gastrula**	P value	0.0014	0.0019	1.0000
		Correction	0.0029	0.0038	1.0000

	<20 cells	20 cells to gastrula
**Control (JIP3105)**	34	69
**G371E (JIP3110)**	59	43
**kcnl-1(0) (JIP3131)**	65	56

			**Control vs G371E**	**Control vs *kcnl-1 (0)***	***kcnl-1 (0)* vs G371E**
**Fisher Exact + Bonferroni correction**	**< 20 cells**	P value	0.0004	0.0020	0.5892
		Correction	0.0009	0.0040	1.0000
	**20 cells to gastrula**	P value	0.0004	0.0020	0.5892
		Correction	0.0009	0.0040	1.0000

Raw data presented in figure 4C

Embryos in utero - I359M

First repetition			Second repetition		
**Control (JIP3105)**	**I359M (JIP3115)**	***kcnl-1(0)* (JIP3131)**	**Control (JIP3105)**	**I359M (JIP3115)**	***kcnl-1(0)* (JIP3131)**
14	27	9	28	42	16
15	29	10	20	40	10
13	32	8	19	41	17
17	27	12	22	36	20
12	23	16	22	41	19
24	33	15	17	40	17
11	29	12	23	35	16
25	20	15	22	36	14
18	23	12	25	45	16
22	26	16	20	37	22
16	19	15	13	38	16
20	21	8	17	43	13
20	33	13	22	47	15
19	22	9	9	35	23
14	27	19	19	40	15
14	23	13	27	43	15
20	27	8	17	48	19
12	20	10	17	39	19
14	29	9	25	33	17
17	26	10	20	37	10
17	26	22	23	43	11
12	14	8	25	30	17
12	20	12	23	28	16
14	33	7	19	39	17
18	25	5	20	37	24
14	33	9	16	43	13
16	19	14	32	42	16
10	38	12	24	36	19
11	26	14	23	35	17
18	30	11	17	41	19

Ordinary One-Way ANOVA P<0.0001; Brown-Forsythe test P<0.0001; Barlett's test P<0.0001

Embryonic stage (%) - I359M

	<20 cells	20 cells to	post gastrula
**Control (JIP3105)**	27	74	
**I359M (JIP3115)**	0	81	20
**kcnl-1(0) (JIP3131)**	62	45	

			**Control vs I359M**	**Control vs *kcnl-1 (0)***	***kcnl-1 (0)* vs I359M**
**Fisher Exact + Bonferroni correction**	**< 20 cells**	P value	0.0000	0.0000	0.0000
		Correction	0.0000	0.0000	0.0000
	**20 cells to**	P value	0.3178	0.0000	0.0000
		Correction	0.9533	0.0000	0.0000
	**post gastrula**	P value	0.0000	1.0000	0.0000
		Correction	0.0000	1.0000	0.0000

Raw data presented in Supplementary Figure 2A

Brood size - I359M

Control (JIP3105)	I359M (JIP3115)	*kcnl-1(0)* (JIP3131)
286	108	267
270	212	305
256	31	266
264	186	304
261	173	239
302	189	262
262	157	298
321	202	292
240	185	259
312	142	237

Kruskal-Wallis test P<0.0001

Raw data presented in Supplementary Figure 2A

Embryos in utero - V530L ZLS3 variants (independent lines)

Control (JIP3105)	V530L (JIP3107)
16	30
15	24
20	37
15	31
20	31
20	33
13	33
11	18
17	29
14	17
14	32
19	31
18	30
16	32
15	33
23	35
21	40
18	34
19	21
19	29
15	34
16	31
16	27
17	29
14	31
14	25
21	23
14	35
10	34
18	35

Mann Whitney test
P value <0.0001

Control (JIP3105)	S436C (JIP3109)
10	41
22	37
15	39
13	43
12	43
14	42
14	46
13	42
9	46
9	41
12	49
11	44
11	33
14	31
10	31
22	46
13	38
13	34
19	31
13	41
14	43
12	35
10	38
11	40
13	29
10	33
9	35
12	40
10	38
12	

Mann Whitney test
P value <0.0001

Raw data presented in Supplementary Figure 2B

Embryonic stage (%) - G350D (independent lines)

First repetition		Second repetition	
Control (JIP3105)	G350D (JIP3128)	Control (JIP3105)	G350D (JIP3128)
15	14	18	17
18	14	12	18
15	20	10	17
11	12	14	15
13	12	18	18
18	12	16	19
17	19	13	11
12	16	16	15
18	11	15	14
18	11	14	16
13	12	16	18
17	15	12	17
11	13	13	17
10	14	16	16
15	15	15	15
14	17	12	14
14	11	10	15
11	13	11	18
13	12	16	16
9	11	11	15
14	12	11	19
13	14	13	17
11	11	19	15
12	12	15	16
14	13	12	19
13	11	12	12
13	16	18	16
15		13	15
13		17	12
9		17	12

Mann Whitney test
P value = 0.1930

First repetition		Second repetition	
Control (JIP3105)	G350D (JIP3129)	Control (JIP3105)	G350D (JIP3129)
12	17	17	18
12	20	19	15
14	17	16	13
12	15	14	18
14	14	24	20
15	19	16	15
10	15	11	22
15	14	15	19
15	14	26	15
15	21	12	25
13	24	16	21
13	17	15	19
15	13	22	13
16	16	18	15
18	32	16	12
14	17	19	14
11	13	14	17
16	19	14	20
13	17	18	16
14	15	14	11
10	19	23	11
15	12	17	19
15	14	17	18
16	10	21	10
16	16	16	15
22	12	15	13
24	19	14	13
15	14	15	18
13	10	20	18
13			17

Mann Whitney test
P value = 0.3307

Raw data presented in Supplementary Figure 2B

Embryonic stage (%) - G350D (independent lines)

	<20 cells	20 cells to	post gastrula
**Control (JIP3105)**	27	67	0
**G350D (JIP3128) line 2**	14	80	0

Fisher Exact + Bonferroni	**Category**	**P value**	**Correction**
	<20 cells	0.0333	0.6650
	20 cells to gastrula	0.6650	0.0665

	<20 cells	20 cells to	post gastrula
**Control (JIP3105)**	38	62	0
**G350D (JIP3129) line 3**	4	110	0

Fisher Exact + Bonferroni	**Category**	**P value**	**Correction**
	<20 cells	0.0000	0.0000
	20 cells to gastrula	0.0000	0.0000

Raw data presented in Supplementary Figure 2C

Embryos in utero - NEDMAB variants (independent lines)

Control (JIP3105)	G362S (JIP3120)	*kcnl-1(0)* (JIP3131)
13	10	13
9	7	10
18	9	8
14	12	10
12	8	13
10	5	6
12	10	17
19	8	10
12	13	8
14	10	10
11	9	19
14	12	8
12	10	8
12	7	10
10	8	11
13	11	11
13	10	8
10	14	9
15	15	14
14	8	8
14	9	8
13	10	10
10	10	13
11	7	8
18	9	15
	10	10
	5	10
	8	11
	9	10

Kruskal-Wallis test
P value<0.0001

Control (JIP3105)	L432P (JIP3126)	kcnl-1(0) (JIP3131)
11	15	11
14	14	10
20	15	11
12	8	12
8	12	9
17	10	17
15	10	9
15	6	11
10	12	8
15	7	8
13	13	10
10	9	6
18	11	7
17	8	8
15	11	8
16	15	10
14	15	12
12	14	8
15	10	11
13	11	12
15	11	21
14	10	10
16	12	14
12	10	14
10	20	9
11	13	17
15	12	13
10	8	9
14	4	15
13	8	
	10	

Kruskal-Wallis test
P value 0.0007

Raw data presented in Supplementary Figure 2C

Embryos in utero - NEDMAB variants (independent lines)

Control (JIP3105)	L388V (JIP3122)	kcnl-1(0) (JIP3131)
19	11	9
16	15	7
13	9	11
15	13	7
16	9	12
13	6	12
12	12	8
20	14	7
13	11	9
11	10	10
14	11	7
10	9	11
15	6	12
15	13	10
14	14	9
15	7	11
20	6	18
22	8	4
24	13	12
22	9	10
21	13	9
15	9	9
18	14	9
13	9	7
18	7	10
17	8	6
15	8	8
16	16	13
15	12	9
14	18	11

Kruskal-Wallis test
P value<0.0001

Control (JIP3105)	I288S (JIP3113)	*kcnl-1(0)* (JIP3131)
13	6	11
16	9	11
12	11	10
13	10	4
14	6	18
13	16	9
12	11	13
13	8	10
16	7	10
11	14	12
14	7	12
17	16	10
10	14	8
13	17	20
12	14	8
20	13	9
16	16	9
17	9	16
15	9	15
14	8	11
16	11	10
11	10	10
14	6	11
19	9	13
20	4	11
14	8	6
15	13	12
11	20	15
16	12	10
12	6	14

Kruskal-Wallis test
P value <0.0001

Raw data presented in Supplementary Figure 2C

Embryos in utero - NEDMAB variants (independent lines)

Control (JIP3105)	G371E (JIP3111)	*kcnl-1(0)* (JIP3131)
19	21	18
14	18	17
21	16	20
13	19	16
19	12	17
13	16	17
13	13	16
24	19	14
19	18	17
18	16	20
18	14	9
17	15	16
16	16	19
23	7	12
22	13	18
21	11	10
12	11	11
24	17	8
11	10	12
16	9	14
21	9	7
19	8	18
22	11	14
18	12	22
17	15	20
16	17	12
19	13	16
18	16	19
21	14	14
23	14	18

Ordinary One-Way ANOVA P<0.0001; Brown-Forsythe test P0.5577; Barlett's test P 0.6298

Control (JIP3105)	I359M (JIP3116)	*kcnl-1(0)* (JIP3131)
18	26	9
14	32	18
13	32	9
18	42	8
12	38	11
18	35	10
13	29	14
14	27	11
17	23	7
12	35	10
16	39	8
15	39	7
15	36	8
10	27	7
18	44	17
13	40	11
19	33	10
21	35	17
19	24	9
13	51	11
15	24	13
11	29	6
16	32	9
15	30	11
12	33	18
19	44	9
12	34	9
18		9
12		11
12		6

Ordinary One-Way ANOVA P<0.0001; Brown-Forsythe test P 0.0003; Barlett's test P<0.0001

Raw data presented in Supplementary Figure 3

Brood size - NEDMAB variants

Control (JIP3105)	G362S (JIP3121)	*kcnl-1(0)* (JIP3131)
250	281	305
122	309	234
271	279	274
299	259	147
254	281	293
314	289	312
262	277	253
268	282	223
323	246	255
290	243	309

Kruskal-Wallis test P=0.8981

Control (JIP3105)	L432P (JIP3124)	*kcnl-1(0)* (JIP3131)
321	298	303
322	294	298
175	299	324
310	293	354
322	305	265
312	339	314
328	350	349
325	361	256
332	334	311
322	284	409

Kruskal-Wallis test P=0.9251

Control (JIP3105)	L388V (JIP3123)	*kcnl-1(0)* (JIP3131)
252		282
169	222	327
337	233	270
231	239	305
255	321	294
272	286	301
289	281	309
365	295	297
	314	346
	365	269

Ordinary One-Way ANOVA
P=0.4123; Brown-Forsythe test
P=0.1358; Barlett's test

Control (JIP3105)	I288S (JIP3114)	kcnl-1(0) (JIP3131)
324	288	183
258	291	286
266	336	283
252	318	282
227	314	305
246	304	240
176	280	231
256	298	316
256	285	310
278	271	321

Ordinary One-Way ANOVA
P=0.0307; Brown-Forsythe test
P=0.3582; Barlett's test

Control (JIP3105)	G371E (JIP3110)	kcnl-1(0) (JIP3131)
286	315	285
314	330	318
271	359	338
293	357	278
264	285	320
285	327	231
367	298	287
325	345	367
301	372	324
258	290	339

Ordinary One-Way ANOVA
P=0.1368; Brown-Forsythe test
P=0.8531; Barlett's test

Appendix 2

In [7]:

```
# Introduction
# This appendix contains the code to perform Fisher's Exact Test and Bonferroni correction,
# with three examples representing different categories or strains that can be analyzed in the Embryonic Stage
# Specifically, the test for I359M, shown in Figure 4C, is reported here for the version of the code handling 3
# The test for the G350D line 2, shown in Figure S2B, corresponds to the version handling 2 categories / 2 stra
# Finally, the test for V530L, shown in Figure 2C, represents the version handling 3 categories / 3 strains.


import numpy as np
import pandas as pd
import scipy.stats as stats
from statsmodels.stats.multitest import multipletests
import matplotlib.pyplot as plt

#1. INPUT DATA
# Create the 2x3 contingency table
data = {
     'Category': ['<20 cells', '20 cells to gastrula', 'post-gastrula'],
   'Control': [27, 74, 0],  # Data for the Control strain
    'I359M': [0, 81, 20],     # Data for the I359M strain
    'kcnl-1(0)': [62, 45, 0]  # Data for the null strain
}

# Convert to DataFrame
df = pd.DataFrame(data)

#  2. CALCULATE P-VALUES FOR EACH COMPARISON
comparisons = [("Control", "I359M"), ("Control", "kcnl-1(0)"), ("I359M", "kcnl-1(0)")]
results = []

for comp1, comp2 in comparisons:
    p_values = []
    for i in range(len(df)):
    # Create a 2x2 contingency table for the comparison
    # We analyze one category at a time, treating it as "in the category" vs. "not in the category".
    # The table consists of:
    # - The count of samples belonging to the category for each strain.
    # - The count of samples not belonging to that category (sum of the other categories).
        table = np.array([
            [df[comp1][i], df[comp2][i]],
            [df[comp1].sum() - df[comp1][i], df[comp2].sum() - df[comp2][i]]
        ])
        # Perform Fisher's Exact Test
        _, p_value = stats.fisher_exact(table, alternative='two-sided')
        p_values.append(p_value)
    # Append results for this comparison
    results.append((comp1, comp2, p_values))


#  3. MULTIPLE COMPARISONS CORRECTION (BONFERRONI)
adjusted_results = []
for comp1, comp2, p_values in results:
    _, p_adjusted, _, _ = multipletests(p_values, method='bonferroni')
    adjusted_results.append((comp1, comp2, p_values, p_adjusted))

# Print the statistical test results
print("Results of Fisher's Exact Test (with Bonferroni correction):")
for comp1, comp2, p_values, p_adjusted in adjusted_results:
    print(f"\nComparison: {comp1} vs {comp2}")
    for i in range(len(df)):
        print(f"  {df['Category'][i]}: p-value = {p_values[i]:.4f}, corrected p-value = {p_adjusted[i]:.4f}")

#  4. CALCULATE PERCENTAGES FOR THE CHART
percent_data = pd.DataFrame({
    'Category': df['Category'],
    'Control': (df['Control'] / df['Control'].sum()) * 100,
    'I359M': (df['I359M'] / df['I359M'].sum()) * 100,
    'kcnl-1(0)': (df['kcnl-1(0)'] / df['kcnl-1(0)'].sum()) * 100
})

#  5. CREATE STACKED BAR CHART
fig, ax = plt.subplots(figsize=(4, 3))

# Set the colors for the categories
colors = ['#3F56A6', '#75B9DE', '#85C66C']

# Define x-axis labels
x_labels = ['Control', 'I359M', 'kcnl-1(0)']

# Stacked bar plots
for i, category in enumerate(df['Category']):
    bottom_values = np.zeros(len(x_labels)) if i == 0 else percent_data.iloc[:i, 1:].sum().values
```

```
    plt.bar(x_labels, percent_data.iloc[i, 1:], bottom=bottom_values, color=colors[i], label=category)

# Add labels, legend, and title
plt.ylabel('Percentage')
plt.title('Distribution of Cells')
plt.legend(title='Category')

# Add labels and title
ax.set_ylabel('Percentage (%)')
ax.set_title('Distribution of Categories by Strain')
ax.set_ylim(0, 100)  # Set the upper limit of the Y-axis to 100%

# Add the legend
ax.legend(title='Developmental Stages', bbox_to_anchor=(1.05, 1), loc='upper left')

# Rotate X-axis labels at 45 degrees
plt.xticks(rotation=45, ha='right')

# Adjust layout for better readability
plt.tight_layout()

# Save the chart as a PDF file for editing in vector graphic software
plt.savefig("graph.pdf", format='pdf', bbox_inches='tight')


# Show the chart
plt.show()
```

```
Results of Fisher's Exact Test (with Bonferroni correction):

Comparison: Control vs I359M
  <20 cells: p-value = 0.0000, corrected p-value = 0.0000
  20 cells to gastrula: p-value = 0.3178, corrected p-value = 0.9533
  post-gastrula: p-value = 0.0000, corrected p-value = 0.0000

Comparison: Control vs kcnl-1(0)
  <20 cells: p-value = 0.0000, corrected p-value = 0.0000
  20 cells to gastrula: p-value = 0.0000, corrected p-value = 0.0000
  post-gastrula: p-value = 1.0000, corrected p-value = 1.0000

Comparison: I359M vs kcnl-1(0)
  <20 cells: p-value = 0.0000, corrected p-value = 0.0000
  20 cells to gastrula: p-value = 0.0000, corrected p-value = 0.0000
  post-gastrula: p-value = 0.0000, corrected p-value = 0.0000
```

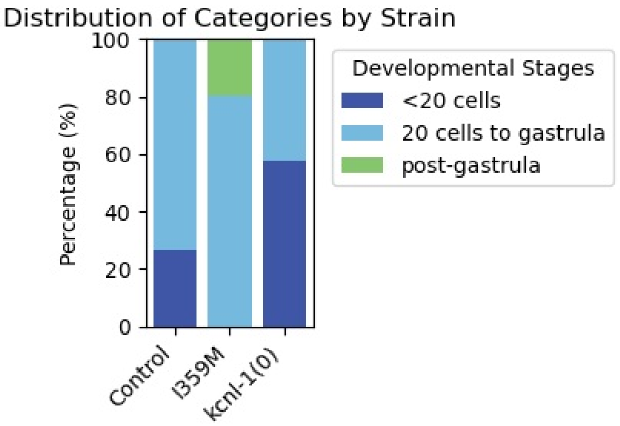

In [ ]:

Loading [MathJax]/jax/output/CommonHTML/fonts/TeX/fontdata.js

In [5]:

```python
# Statistical libraries #
import numpy as np
import pandas as pd
import scipy.stats as stats
from statsmodels.stats.multitest import multipletests
import matplotlib.pyplot as plt

# 1. INPUT DATA
# Define the dataset containing categories and their respective counts for Control and G350D strains.
data = {
    'Category': ['<20 cells', '20 cells to gastrula'],  # Developmental stages
    'Control': [27, 67],  # Observations for the Control strain
    'G350D': [14, 80]     # Observations for the G350D strain
}

# Convert the dictionary into a pandas DataFrame for easier data manipulation
df = pd.DataFrame(data)

# 2. PERFORM FISHER'S EXACT TEST FOR EACH CATEGORY
p_values = []  # List to store p-values
for i in range(len(df)):
    # Create a 2x2 contingency table for Fisher's Exact Test
    # Therefore, we analyze one category at a time, treating it as "in the category" vs. "not in the category".
    # The table consists of:
    # - The count of samples belonging to the category for each strain.
    # - The count of samples not belonging to that category (sum of the other categories).
    table = np.array([
        [df['Control'][i], df['G350D'][i]],  # Samples in the current category for both strains
        [df['Control'].sum() - df['Control'][i], df['G350D'].sum() - df['G350D'][i]]  # Samples in other catego
    ])

    # Perform Fisher's Exact Test (two-sided)
    _, p_value = stats.fisher_exact(table, alternative='two-sided')
    p_values.append(p_value)  # Store the p-value

# 3. MULTIPLE COMPARISONS CORRECTION (BONFERRONI)
# Adjust p-values using the Bonferroni correction to account for multiple comparisons
_, p_adjusted, _, _ = multipletests(p_values, method='bonferroni')

# Print the statistical test results
print("Results of Fisher's Exact Test (with Bonferroni correction):")
for i in range(len(df)):
    print(f"{df['Category'][i]}: p-value = {p_values[i]:.4f}, corrected p-value = {p_adjusted[i]:.4f}")

# 4. CALCULATE PERCENTAGES FOR THE CHART
# Convert absolute counts into percentages to visualize the proportion of each category

df['Control_percent'] = (df['Control'] / df['Control'].sum()) * 100

df['G350D_percent'] = (df['G350D'] / df['G350D'].sum()) * 100

# 5. CREATE STACKED BAR CHART
# Initialize a figure with specified dimensions
fig, ax = plt.subplots(figsize=(4, 3))

# Define colors for each category in the stacked bar chart
colors = ['#3F56A6', '#75B9DE']

# Create stacked bars for 'Control' group
top_control = ax.bar('Control', df['Control_percent'][0], color=colors[0], label=df['Category'][0])
bottom_control = ax.bar('Control', df['Control_percent'][1], bottom=df['Control_percent'][0], color=colors[1], 

# Create stacked bars for 'G350D' group
top_g350d = ax.bar('G350D', df['G350D_percent'][0], color=colors[0])
bottom_g350d = ax.bar('G350D', df['G350D_percent'][1], bottom=df['G350D_percent'][0], color=colors[1])

# Set labels and title
ax.set_ylabel('Percentage (%)')
ax.set_xlabel('Strain')
ax.set_title('Distribution of Categories by Strain (Control vs G350D)')
ax.set_ylim(0, 100)  # Set Y-axis limits to 100% to represent proportions correctly

# Add a legend to distinguish categories
ax.legend(title='Developmental Stages', bbox_to_anchor=(1.05, 1), loc='upper left')

# 6. SAVE THE CHART AS A PDF FILE
# Save the chart for further editing in vector graphic software
plt.savefig("graph.pdf", format='pdf', bbox_inches='tight')

# Show the chart
plt.tight_layout()
plt.show()
```

```
Results of Fisher's Exact Test (with Bonferroni correction):
<20 cells: p-value = 0.0333, corrected p-value = 0.0665
20 cells to gastrula: p-value = 0.0333, corrected p-value = 0.0665
```

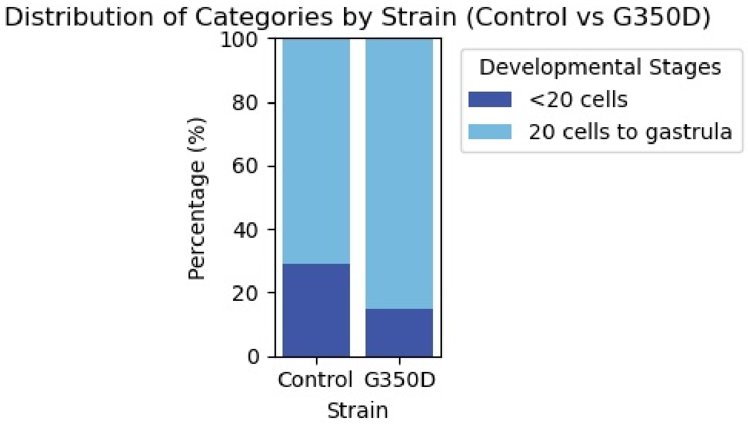

In [ ]:

Loading [MathJax]/jax/output/CommonHTML/fonts/TeX/fontdata.js

In [3]:
```
import numpy as np
import pandas as pd
import scipy.stats as stats
from statsmodels.stats.multitest import multipletests
import matplotlib.pyplot as plt

# === 1. INPUT DATA ===
# Create the 2x3 contingency table
data = {
    'Category': ['<20 cells', '20 cells to gastrula', 'post-gastrula'],
    'Control': [34, 68, 0],  # Data for the Control strain
    'V530L': [0, 78, 25]     # Data for the V530L strain
}

# Convert to DataFrame
df = pd.DataFrame(data)

# === 2. PERFORM FISHER'S EXACT TEST FOR EACH CATEGORY ===
p_values = []
for i in range(len(df)):
    # Create a 2x2 table for each category
    table = np.array([[df['Control'][i], df['V530L'][i]],
                      [df['Control'].sum() - df['Control'][i], df['V530L'].sum() - df['V530L'][i]]])
    # Perform Fisher's Exact Test
    _, p_value = stats.fisher_exact(table, alternative='two-sided')
    p_values.append(p_value)

# Adjust the p-values using Bonferroni correction
_, p_adjusted, _, _ = multipletests(p_values, method='bonferroni')

# Print the results
print("Results of Fisher's Exact Test (with Bonferroni correction):")
for i in range(len(df)):
    print(f"{df['Category'][i]}: p-value = {p_values[i]:.4f}, corrected p-value = {p_adjusted[i]:.4f}")

# === 3. CREATE STACKED BAR CHART ===
# Calculate percentages for the stacked bar chart
df['Control_percent'] = (df['Control'] / df['Control'].sum()) * 100
df['V530L_percent'] = (df['V530L'] / df['V530L'].sum()) * 100

# Create the chart
fig, ax = plt.subplots(figsize=(4, 3))

# Set the colors for the categories
colors = ['#3F56A6', '#75B9DE', '#85C66C']

# Create stacked bars for 'Control'
ax.bar('Control', df['Control_percent'][0], color=colors[0], label=df['Category'][0])
ax.bar('Control', df['Control_percent'][1], bottom=df['Control_percent'][0], color=colors[1], label=df['Category
ax.bar('Control', df['Control_percent'][2], bottom=df['Control_percent'][0] + df['Control_percent'][1], color=co

# Create stacked bars for 'S436C'
ax.bar('V530L', df['V530L_percent'][0], color=colors[0])
ax.bar('V530L', df['V530L_percent'][1], bottom=df['V530L_percent'][0], color=colors[1])
ax.bar('V530L', df['V530L_percent'][2], bottom=df['V530L_percent'][0] + df['V530L_percent'][1], color=colors[2]

# Add labels and title
ax.set_ylabel('Percentage (%)')
ax.set_xlabel('Strain')
ax.set_title('Distribution of Developmental Stages by Strain (Control vs V530L)')
ax.set_ylim(0, 100)  # Set the upper limit of the Y-axis to 100%

# Add the legend
ax.legend(title='Developmental Stages', bbox_to_anchor=(1.05, 1), loc='upper left')

# Save the chart as a PDF file for editing in vector graphic software
plt.savefig("graph.pdf", format='pdf', bbox_inches='tight')

# Show the chart
plt.tight_layout()
plt.show()
```

```
Results of Fisher's Exact Test (with Bonferroni correction):
<20 cells: p-value = 0.0000, corrected p-value = 0.0000
20 cells to gastrula: p-value = 0.1673, corrected p-value = 0.5018
post-gastrula: p-value = 0.0000, corrected p-value = 0.0000
```

Distribution of Developmental Stages by Strain (Control vs V530L)

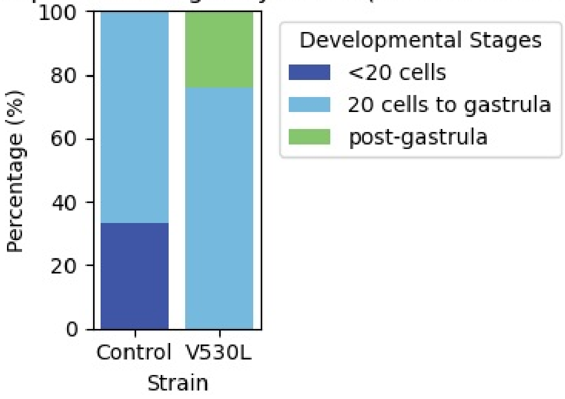

In [ ]:

Loading [MathJax]/jax/output/CommonHTML/fonts/TeX/fontdata.js
